# Nanoscale Failure Mechanism and Nanoengineering Modification Strategies of Layered NCM Cathodes

**DOI:** 10.3390/nano16171102

**Published:** 2026-09-01

**Authors:** Rui Xu, Xue Liu, Yi Wang, Jean-Jacques Gaumet, Chaojiang Niu, Wen Luo

**Affiliations:** 1 School of Physics and Mechanics, Wuhan University of Technology, Wuhan 430070, China; 2 State Key Laboratory of Advanced Technology for Materials Synthesis and Processing, School of Materials Science and Engineering, Wuhan University of Technology, Wuhan 430070, China; 3 School of Materials Science and Engineering, Zhengzhou University, Zhengzhou 450001, China

**Keywords:** nanoscale, NCM, failure mechanism, nanoengineering, modification strategy

## Abstract

Layered cathodes (LiNi_x_Co_y_Mn_z_O_2_, NCM) have emerged as critical materials for batteries and energy storage fields by virtue of their high energy density. However, NCM materials undergo rapid performance degradation and severe capacity fading under harsh conditions of long-term cycling and high voltage. Currently, research regarding spent NCM materials mainly concentrates on failure analysis and modification processes at the macroscopic scale. Nevertheless, the failure mechanisms of NCM, the intrinsic processes during repair and modification, and the fundamental origins of performance improvement are generally embedded in structural evolution at the nanoscale or even atomic scale. This review first discusses the failure mechanisms of NCM. Particularly, the main content focuses on lattice distortion and layered structural instability at the lattice level, migration of nanoscale species together with performance degradation induced by side reactions at the interface level, and generation of nanocracks at the particle level. Moreover, this paper reviews the characterization methods applied at the nanometer scale, and two modification strategies are summarized, namely nanoscale coating and elemental doping. It is expected to provide theoretical references and technical insights for constructing efficient and controllable targeted modification strategies of layered NCM cathodes and developing high-performance ternary cathode materials.

## 1. Introduction

Recently, layered LiNi_x_Co_y_Mn_z_O_2_ (NCM) cathode materials have rapidly become the dominant option for power and energy storage batteries by virtue of their high energy density and excellent low-temperature electrochemical properties [1,2]. However, with the continuous growth in the installed capacity of power batteries and the gradual retirement of early-service lithium-ion batteries, the quantity of spent NCM batteries is showing an explosive growth trend [3,4]. Therefore, in-depth analysis of battery failure mechanisms has become increasingly critical. In particular, investigating nanoscale microscopic failure mechanisms is of great significance for optimizing structural properties.

The underlying failure mechanisms, inherent modification processes, and fundamental sources of performance improvement for NCM cathodes originate from structural variations occurring at nanometer or even atomic dimensions [5,6,7]. Under long-term cycling or extreme operating conditions, a series of complex nanoscale structural degradations occur inside NCM materials, including aggravated Li/Ni cation mixing, dissolution, and generation and propagation of microcracks [8,9,10,11]. However, macroscopic characterizations can reflect the average structural information of NCM lattices and rapidly acquire the electrochemical performances of batteries, yet they fail to precisely characterize the structural damage of NCM at the nanoscale [12]. Conversely, nanoscale characterization techniques enable precise acquisition of localized defects in NCM, such as regional lattice distortion and microcrack formation [13]. Therefore, it is of great significance to explore the fundamental origins of failure mechanisms in NCM materials via nanoscale characterization. However, with in-depth research on the nanoscale failure mechanisms of NCM, researchers have revealed that the forms of electrochemical performance degradation induced by distinct failure mechanisms differ significantly [14,15]. To effectively eliminate microscale defects in NCM, appropriate modification strategies must be adopted to optimize their electrochemical performance in batteries. Furthermore, widely adopted effective remediation and upgrading strategies include interface engineering [16,17], lattice doping [18,19,20], surface coating [21,22,23], and so on, whose underlying mechanisms also operate at the nanoscale.

Relevant existing studies covering failure mechanisms, modification strategies, and microscopic characterizations remain scattered, lacking systematic collation and summary. Therefore, this paper systematically reviews and analyzes the failure mechanisms, modification strategies, and characterization techniques of NCM cathodes from a nanoscale perspective. This article systematically elaborates on the following contents: (1) nanoscale failure mechanisms of NCM materials during cycling, including lattice distortion and layered structural instability at the lattice scale, nanoscale mass transfer and degradation induced by side reactions at the interfacial scale, as well as generation of nanocracks at the particle scale. (2) Advanced characterization techniques applicable to observing, analyzing, and characterizing the aforementioned nanoscale structural and chemical variations. (3) Nanoscale modification strategies for performance restoration and enhancement, including nanoscale coating modification, nanoscale doping modification, and synergistic dual modification strategies. This paper analyzes failure mechanisms, summarizes characterization techniques, and reviews modification approaches from a nanoscale perspective. From the perspective of nanoscale failure mechanisms, this work is expected to provide theoretical references and technical insights for designing novel nanoengineering modification schemes of NCM cathodes and optimizing their electrochemical stability.

## 2. Nanoscale Failure Mechanisms of Layered NCM Cathodes

The performance decay of layered NCM cathodes under long-term cycling and high voltage essentially originates from multi-scale structural degradation spanning atomic, nanoscale, and microscopic dimensions [24]. However, such structural degradation is confined within a scale ranging from several to hundreds of nanometers and thus cannot be detected via macroscopic measurements [25,26,27]. In contrast, nanoscale analysis can achieve sub-nanometer to nanometer resolution, enabling direct observation and precise phase identification at the single-particle, local interfacial, and atomic-layer scales [28,29]. Consequently, this chapter will address three types of failure mechanisms: lattice distortion and layered structural instability at the lattice scale, nanocrack generation at the particle scale, and nanomass migration as well as deterioration triggered by side reactions at the interfacial scale.

### 2.1. Lattice Distortion and Layered Structural Instability at Lattice Scale

**Figure 1:**
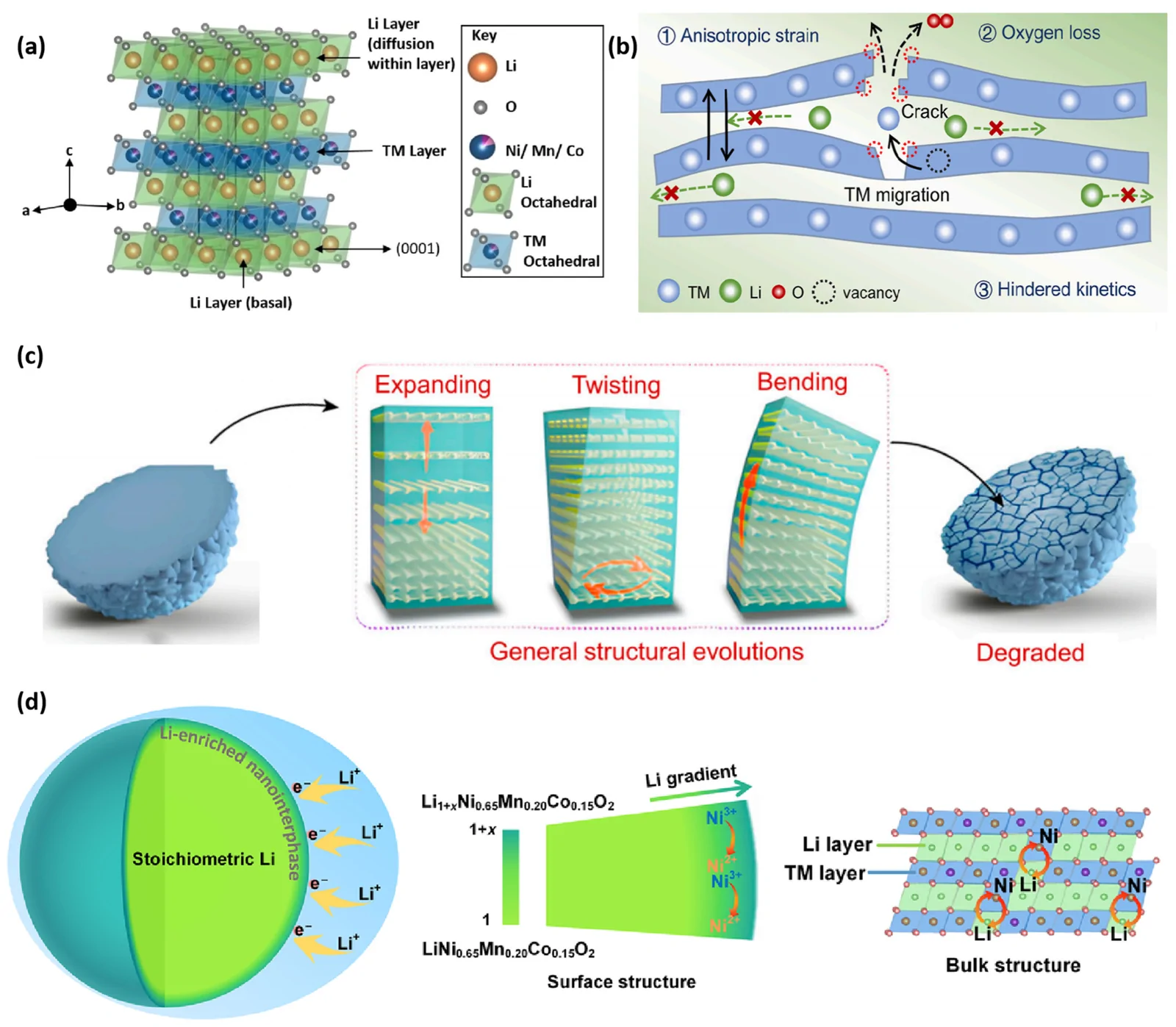
(**a**) Layered structure of NCM [31]. Copyright 2024, John Wiley & Sons. (**b**) Schematic illustration of the failure mechanism of degraded NCM [42]. Copyright 2026, Elsevier. (**c**) Schematic diagram showing the failure mechanism of lattice rotation in NCM induced by radial stress [50]. Copyright 2024, American Association for the Advancement of Science. (**d**) Formation of lithium-enriched gradient interfacial structure and Li–Ni cation mixing in NCM upon cycling [56]. Copyright 2021, American Chemical Society.

NCM cathode materials belong to the hexagonal crystal system with an R-3m space group [30]. They adopt an α-NaFeO_2_-type crystal structure (Figure 1a), in which LiO_6_ and TMO_6_ octahedra are alternately stacked along the c-axis [31]. The stable stacking configuration is primarily maintained by Li–O bonds and van der Waals forces. During the charging process of NCM cathodes, lithium ions (Li^+^) are extracted from the lithium interlayer. The discrepancy in Li^+^ extraction rate between the surface and internal grains induces extensive cleavage of Li–O coordination bonds, thereby triggering lattice-level structural degradation [32,33,34]. Furthermore, such failures can be classified into interlayer slide and slip, lattice rotation, and layered structural instability according to their generation causes and structural consequences.

By analyzing the lattice structure of degraded NCM cathodes, several types of nanoscale interlayer slide and slip are identified [35]. During cycling, the concentration gradient of Li^+^ induces radial stress within the NCM lattice, which further triggers interlayer slide and slip between transition metal layers [36,37,38]. Localized slide and slip in layered NCM cathodes are primarily realized via the in-plane migration of transition metal (TM) ions, and this structural evolution is significantly affected by oxygen vacancies [39]. Oxygen vacancies in NCM grains are closely correlated with the kinetic energy barrier of TM migration [40]. Increased oxygen vacancies facilitate the penetration of TM ions through the oxygen layers and subsequent occupation of Li sites (Figure 1b), thereby accelerating the distortion and stacking-fault evolution of two-dimensional layered platelets [41,42]. Furthermore, based on the nanoscale strain-step phenomenon occurring during the charge–discharge process, the layered slip behavior is further classified into discrete slip and continuous slip [43].

Furthermore, nanoscale lattice rotations can also be detected in degraded NCM cathodes. Lattice rotation refers to the lattice distortion phenomenon in which the crystal lattice undergoes overall angular deflection [44]. Nanoscale Multicrystal Rocking Curve (MCRC) of lattice rotation reveals that interlayer anisotropic stress serves as the dominant driving force for lattice rotation [45]. In the charging process, uneven Li^+^ deposition and non-uniform reactions between particles give rise to localized lattice stress. To relieve lattice stress, lattice rotation occurs in NCM. During discharge, Li^+^ reintercalates into the lattice framework of NCM [46,47,48]. However, this process can only eliminate most of the lattice strain, while the structural defects and lattice distortion induced by lattice rotation cannot be removed (Figure 1c) [49,50]. Furthermore, continuous accumulation of lattice rotation exacerbates plastic deformation and mechanical damage of NCM during cycling, which hinders Li^+^ diffusion and thereby constitutes an irreversible capacity-fading mechanism [51,52].

In addition, degraded NCM cathodes exhibit cation mixing arising from nanoscale interlayer migration of Li^+^ and Ni^2+^, which further triggers degradation characterized by destabilized layered frameworks of NCM. Due to the extremely similar ionic radii of divalent nickel ions (Ni^2+^, 0.069 nm) and lithium ions (Li^+^, 0.076 nm), Ni^2+^ can cross an extremely low diffusion energy barrier and undergo nanoscale interlayer migration under deep delithiation conditions. As a result, Ni^2+^ occupies the 3b Li-site vacancies, thereby inducing cation mixing (Figure 1d) [53,54,55,56]. Moreover, cation mixing aggravates the structural instability of NCM cathodes, resulting in atomic-scale lattice slides and antisite defects inside grains [57]. Furthermore, these defects not only disrupt the intrinsic Li^+^ diffusion pathways but also trigger defect chain reactions (DCR), which induce rock-salt phase transformation and consequent electrochemical performance degradation [58,59].

### 2.2. Nanoscale Mass Transfer and Degradation Induced by Side Reactions at Interfacial Scale

**Figure 2:**
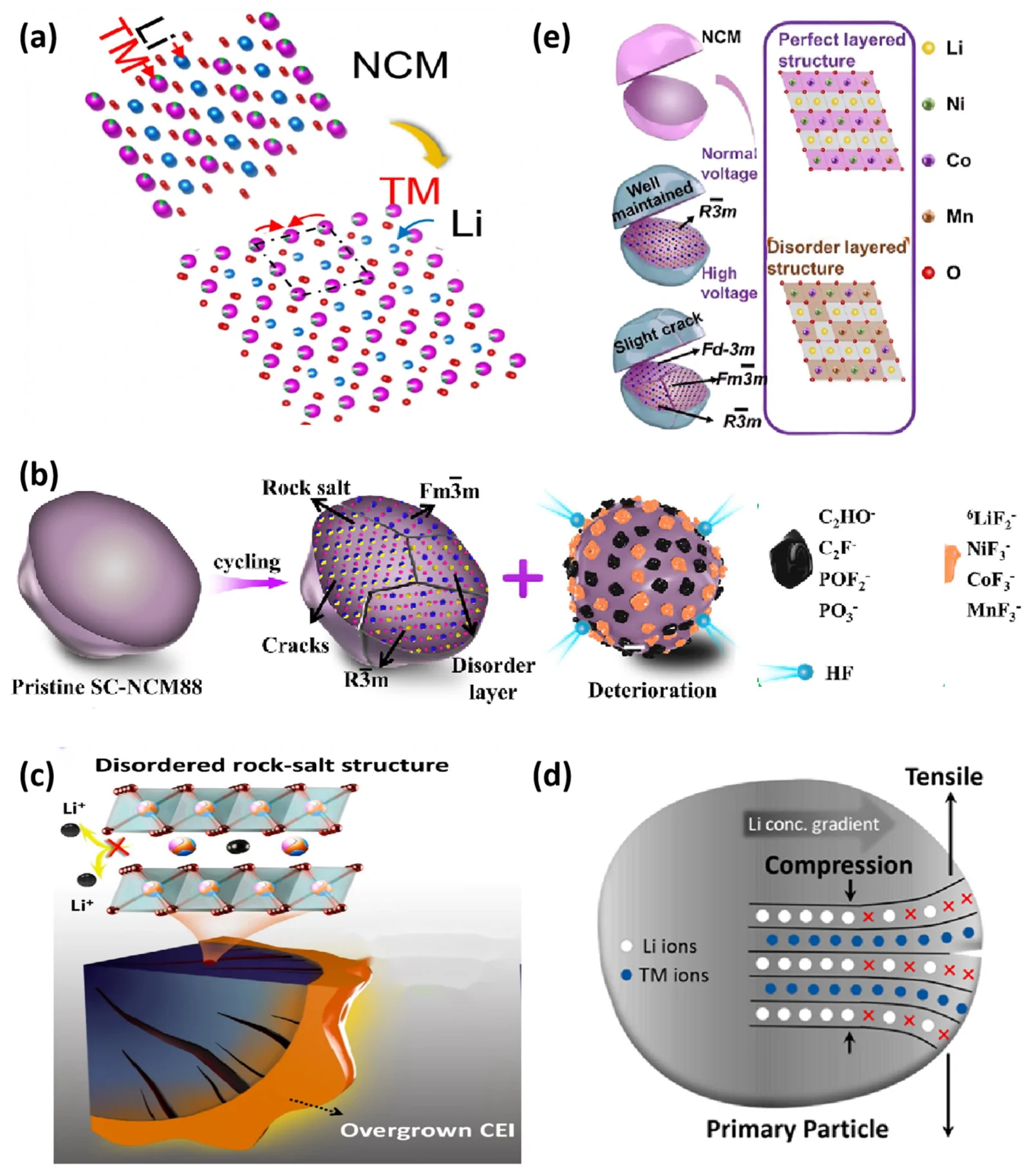
(**a**) Phase transformation of layered NCM from layered structure to spinel phase [63]. Copyright 2022, Springer Nature. (**b**) Schematic illustration showing the evolution of pristine single-crystal LiNi_0.88_Co_0.09_Mn_0.03_O_2_ (SC-NCM88) particles upon long-term cycling, including the generation of Rock-Salt phase and internal cracks as well as the formation of CEI film on the surface [72]. Copyright 2021, Springer Nature. (**c**) Internal structural evolution and surface CEI film growth of NCM during long-term cycling [97]. Copyright 2022, Springer Nature. (**d**) Generation of radial stress in NCM upon cycling [104]. Copyright 2025, American Chemical Society. (**e**) Layered-to-Rock-Salt phase transition of layered NCM upon cycling, which ultimately induces intra-particle microcracks [117]. Copyright 2024, KeAi Communications Co., Ltd.

There are two manifestations of interfacial degradation in NCM cathodes. The first one refers to the surface reconstruction layer (SRL) formed by the irreversible conversion of the layered structure into rock-salt phase (Figure 2a) [60,61,62,63]. The SRL exhibits high-resistance characteristics, and the phase transition process simultaneously releases reactive oxygen species, thereby inducing continuous electrolyte decomposition [64,65]. The second form corresponds to the growth of the cathode–electrolyte interphase (CEI) film [66,67]. The CEI film originates from chemical and electrochemical reactions occurring between the electrolyte and the surface of cathode active materials [68]. The growth of the CEI film continuously consumes the electrolyte, which induces the permanent loss of active lithium and increases the interfacial charge-transfer resistance [69,70,71]. Therefore, the formation of SRL and CEI films is one of the core causes responsible for the degradation of NCM cathodes (Figure 2b) [72].

#### 2.2.1. Formation Mechanism of Interfacial Rock-Salt Phase

The Rock-Salt phase is a NaCl-type defective phase generated by lattice oxygen release and TM–Li^+^ cation mixing during the cycling of NCM cathodes [73]. This phase mainly accumulates on the surface layer of cycled NCM particles and inner walls of microcracks, with a typical thickness of several nanometers [74]. Furthermore, it mostly exists as a continuous thin shell coating particle surfaces, while isolated island-like and irregular sheet-like Rock-Salt domains can also be observed inside particles [75].

Under highly delithiated conditions, massive extraction of Li^+^ triggers simultaneous oxidation of Ni^2+^ to Ni^3+^ and Ni^3+^ to Ni^4+^ within NCM materials [76]. At high potentials, the energy levels of the Ni^4+^/Ni^3+^ redox couple lie close to the top of the O 2p band, which weakens the stability of metal–oxygen bonds. This further triggers lattice oxygen release from the surface layer of NCM and produces irreversible oxygen defects on the surfaces of primary particles [77,78,79]. Oxygen defects reduce the exchange energy barrier between Li^+^ and Ni^2+^, thus exacerbating cation mixing within particles [80]. In addition, Li/Ni mixing continuously disrupts the ordered layered framework and increases the structural disorder of the surface layer. Meanwhile, it induces the layered phase on NCM particle surfaces to gradually transform into the Rock-Salt phase through a disordered spinel intermediate [81,82,83]. The dense crystal structure of the Rock-Salt phase three-dimensionally blocks Li^+^ diffusion pathways. Meanwhile, this phase preferentially accumulates at the material surface and interfaces, severely hindering charge transport and ultimately deteriorating the electrochemical performance of NCM cathodes [84,85,86].

#### 2.2.2. Degradation Induced by CEI Film Growth

The cathode–electrolyte interphase (CEI) film is a passivation interphase film covering the surface of layered cathode particles, which consists of oxidative decomposition products of the electrolyte [87]. A typical CEI possesses a layered nanostructure composed of an outer organic-rich layer and an inner inorganic-rich layer. Among them, inorganic components include lithium fluoride, salt degradation products (Li_x_PO_y_F_z_), and nickel fluoride (NiF_2_) derived from NCM. In contrast, organic compounds mainly consist of electrolyte decomposition products such as C_x_H_y_O_z_ and ROLi [88,89]. The thickness of CEI films generally ranges from tens of nanometers and can even reach several hundred nanometers in some cases [90].

Under normal cycling conditions, a thin and compact CEI film forms on the surface of NCM particles [91,92]. A thin and compact CEI film can improve the interfacial stability of cathodes, suppress the phase transition of the Rock-Salt structure, accelerate the Li^+^ diffusion rate, enhance capacity retention, and alleviate the fracture of NCM particles over cycling [93,94]. However, with the increase in cycling numbers, the CEI film undergoes continuous thinning and thickening evolution and gradually grows thicker, ultimately forming a thick and loose CEI layer on the NCM surface [95]. In contrast, excessively thick CEI films significantly increase the transport resistance for lithium ions crossing the interphase (Figure 2c), leading to aggravated battery polarization and deteriorated rate performance [96,97]. Moreover, the accumulation of numerous insulating byproducts within the thick film further elevates the interfacial impedance continuously, exacerbating the irreversible capacity loss and severely deteriorating the long-cycle lifespan of batteries [98,99].

### 2.3. Generation of Nanocracks at Particle Scale

Studies have revealed that irreversible interlayer sliding carries the risk of further inducing microcracks. In addition, the formation mechanism of nanoscale microcracks can be investigated via interlayer sliding behavior [100,101]. Under high-voltage charge–discharge conditions, nickel-rich NCM materials undergo a discontinuous H2-to-H3 phase transition in the late stage of deep delithiation, generating substantial mechanical strain inside particles (Figure 2d) [102,103,104]. This triggers interlayer sliding within NCM grains to release accumulated stress. Moreover, with the continuous accumulation of interlayer sliding, high-density slide zones and localized lattice distortion emerge [105]. Furthermore, nanoscale stress singularities are eventually formed at slip bands, which serve as preferential nucleation sites for microcracks and consequently initiate the generation of nanoscale microcracks [106,107,108]. Microcracks exert different effects on materials depending on their formation locations. Microcracks located near the interlayer sliding regions exhibit a typical asymmetric morphology [109]. These cracks originate from the parallel splitting of adjacent transition metal layers, which causes no damage to the grain matrix and is reversible, enabling self-repair during subsequent cycling [110]. In contrast, microcracks within interlayer sliding regions induce substantial material degradation. Such degradation inhibits crack self-repair and further triggers the deterioration of mechanical properties inside cathode grains [111].

In addition, electrolyte stability also affects the generation of microcracks inside NCM grains [112]. At high delithiation stages, the interaction between electrolyte decomposition products and the surface of NCM particles consumes and releases lattice oxygen, disrupting the TMO_2_ framework and reducing Ni^3+^ and Ni^4+^ to Ni^2+^. Meanwhile, Ni^2+^ migrates to lithium sites, driving the transformation of the layered structure into an inactive Rock-Salt phase [113,114]. Due to the significant mismatch in expansion coefficients between the Rock-Salt phase and layered structure, grain-boundary stress emerges at the phase interfaces, thereby nucleating nanoscale microcracks on the surfaces and grain boundaries of NCM particles (Figure 2e) [115,116,117]. Therefore, microcracks arise from the synergistic accumulation of strain induced by lattice defects and interfacial reactions at NCM particle surfaces [118]. In addition, microcracks can be classified into intragranular cracks, intergranular cracks, and transgranular cracks based on their locations. Intragranular cracks propagate entirely within individual grains without crossing grain boundaries. Intergranular cracks extend along grain boundaries with their trajectories closely fitting the grain edges and rarely penetrating into grain interiors. However, transgranular cracks directly cross grain boundaries, penetrate multiple grains, and cut through the grain matrix.

## 3. Nanoscale Characterization Techniques for Failure Analysis and Modification of NCM Cathodes

Adopting appropriate characterization techniques lays the foundation for exploring and elucidating the failure mechanisms of NCM cathodes. The aforementioned failure mechanisms take place at the nanoscale, which necessitates the utilization of advanced characterization techniques with nanometer or even atomic-level resolution for analysis [119,120,121,122]. This chapter classifies nanoscale characterization methods into two major categories: ex situ characterization and in situ dynamic characterization.

### 3.1. Nanoscale Characterization Techniques for Microstructure and Composition

The ex situ characterization system enables the analysis of intrinsic microscopic features of electrode particles from the nanometer down to the atomic scale under offline steady-state conditions [123]. Further, it can compensate for the deficiency that macroscopic characterization fails to capture information regarding localized heterogeneous degradation [124,125]. This chapter divides all techniques into three categories by characterization function and scale: high-resolution electron microscopy for morphology and atomic structure, micro-region characterization for elemental composition and valence state, and non-destructive three-dimensional and cross-sectional microstructure characterization. Furthermore, these three types of characterization techniques perform multi-level ex situ analysis covering two-dimensional (2D) morphology, elemental valence states, and three-dimensional (3D) structures and provide comprehensive nanoscale experimental evidence for clarifying the local microscopic failure mechanisms of materials.

#### 3.1.1. High-Resolution Electron Microscopy for Morphology and Atomic Structure Characterization

The ex situ nanoscale characterization system centered on transmission electron microscopy (TEM) enables sub-nanometer spatial resolution observation. High-resolution electron microscopy for morphological and atomic structure characterization is centered on TEM and STEM imaging techniques, featuring ultrahigh spatial resolution. In addition, this technique can directly acquire atomic-scale morphology, lattice fringes, and atomic arrangement defects at grain boundaries of NCM materials [126]. The mainstream approaches of this characterization technique include high-resolution transmission electron microscopy (HRTEM) and fast Fourier transform (FFT) under TEM, as well as Annular Dark-Field Scanning Transmission Electron Microscopy (ADF-STEM), High-Angle Annular Dark-Field Scanning Transmission Electron Microscopy (HAADF-STEM), and other modes implemented based on double aberration-corrected scanning transmission electron microscopes [127,128,129].

**Figure 3:**
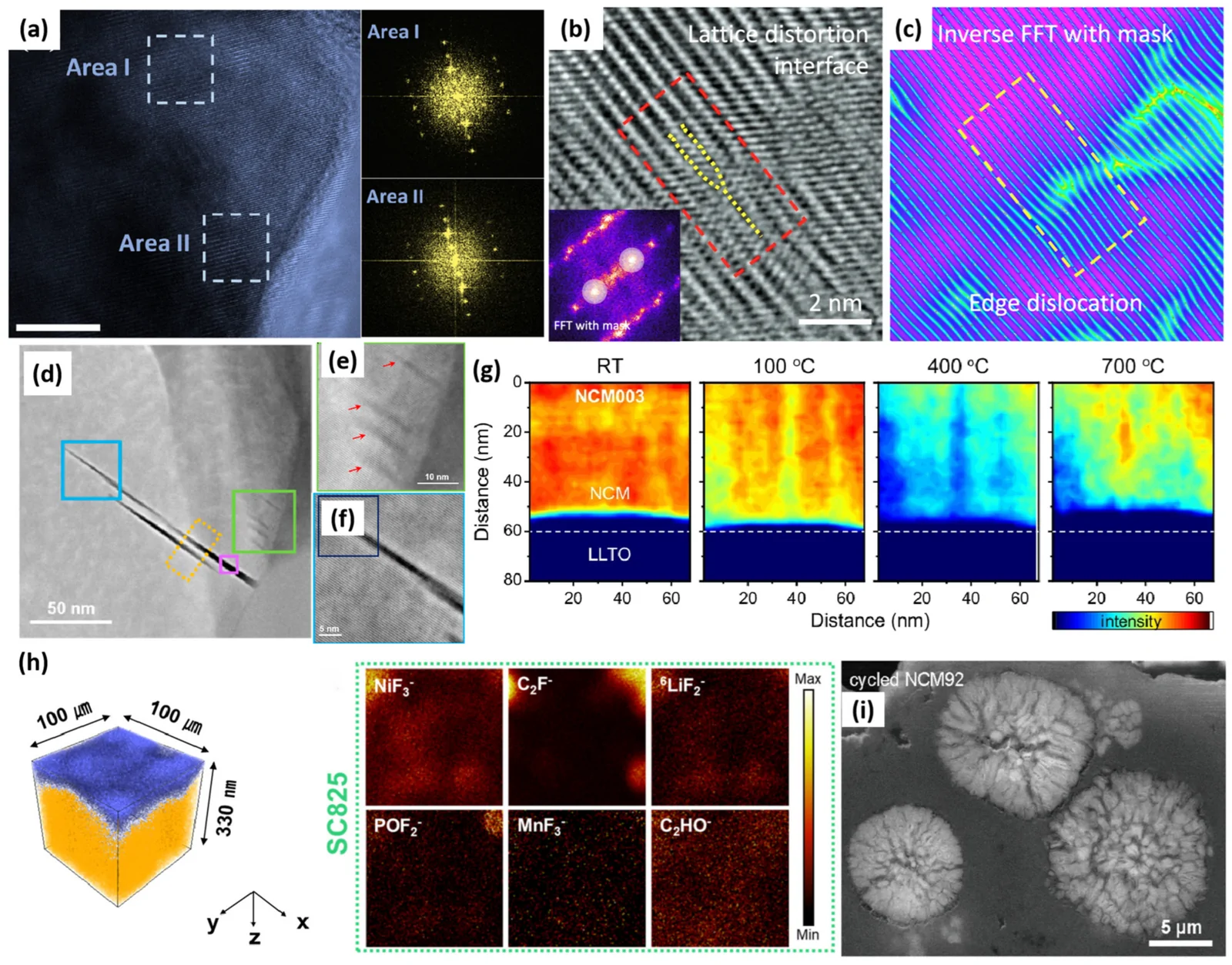
(**a**) TEM image of NCM622 and the corresponding FFT pattern derived from the TEM image [132]. Copyright 2024, Springer Nature. (**b**) HRTEM image and (**c**) its FFT pattern showing an edge-dislocation defect at the lattice-distorted interface of NCM [136]. Copyright 2025, Springer Nature. (**d**) HAADF-STEM image of the 2C-charged NMC811 cathode with several intragranular nanocracks. (**e**,**f**) Enlarged HAADF-STEM images of regions marked in (**d**) [104]. Copyright 2025, American Chemical Society. (**g**) STEM images of NCM003 during heating and the corresponding Li EELS mapping [153]. Copyright 2024, Springer Nature. (**h**) Three-dimensional rendered TOF-SIMS chemical imaging of SC825 (Single crystal Ni-rich LiNi_0.92_Co_0.04_Mn_0.04_O_2_ sintered at 825 °C) and PC (polycrystalline Ni-rich LiNi_0.92_Co_0.04_Mn_0.04_O_2_) electrodes after 100 cycles within the voltage range of 3.0–4.3 V, together with surface composition (Ni and NiF_3_^−^) of SC825 [155]. Copyright 2024, John Wiley & Sons, Inc. (**i**) FIB-SEM cross-sectional image of NCM92 revealing the ubiquitous intra-particle crack network in cycled NCM particles, with a characteristic spacing of 100–300 nm [166]. Copyright 2026, John Wiley & Sons, Inc.

As a general tool for microscopic characterization, conventional TEM achieves a resolution limit at the nanometer and sub-nanometer scales. Furthermore, this technique enables direct acquisition of nanomorphological information of electrode powder particles, including particle size, pore defects, and thickness of surface coating layers (Figure 3a) [130,131,132]. Meanwhile, it possesses the merits of simple sample preparation procedures, high testing efficiency, and a wide application range. HRTEM is employed to characterize fine lattice structures such as interplanar spacings and crystal planes, and is frequently combined with FFT for correlative analysis [133]. Moreover, FFT transforms real-space lattice images into reciprocal-space diffraction spots, thereby enabling the identification of lattice distortion and impurity phases. The combination of HRTEM and FFT serves as a widely adopted ex situ characterization method for analyzing the crystal structure of layered NCM materials (Figure 3b,c) [134,135,136]. Furthermore, this system features the merits of intuitive lattice information and lower testing costs compared with atomic-resolution STEM facilities. The double aberration-corrected STEM constitutes the core hardware platform for atomic-resolution static characterization. Meanwhile, this electron microscope has high-resolution imaging modes, including ADF-STEM, HAADF-STEM, and Differential Phase-Contrast Scanning Transmission Electron Microscopy (DPC-STEM) [137,138,139]. Among them, ADF-STEM is mainly utilized to observe crystal morphology and light-element coating layers, which is suitable for the characterization of carbon coatings and thin amorphous byproduct layers [140,141]. HAADF-STEM allows direct visualization of cation mixing, surface segregation of transition metals, and core–shell grain structures, thereby enabling qualitative analysis of elemental spatial distribution at single-atom resolution (Figure 3d–f) [104,142,143]. DPC-STEM can directly detect the localized built-in electric fields both inside and outside NCM particles, and further perform quantitative analysis on failure mechanisms such as lattice strain and slide slip [144].

#### 3.1.2. Micro-Region Characterization of Elemental Composition and Valence State Structure

Characterization of microscale elemental composition and valence state structures covers a series of spectroscopic techniques, including Energy-Dispersive X-ray Spectroscopy (EDS), Electron Energy-Loss Spectroscopy (EELS), Time-of-Flight Secondary Ion Mass Spectrometry (TOF-SIMS), and Extended X-ray Absorption Fine Structure (EXAFS) [145,146,147]. These characterization techniques allow qualitative and quantitative elemental determination, elemental profiling of three-dimensional interfaces, and elucidation of metal valence states and coordination environments [148]. Furthermore, they compensate for the limitations of conventional bulk characterization in elucidating chemical information of microregions. Most importantly, they serve as critical characterization tools for correlating the microchemical evolution of NCM with electrochemical degradation and guiding the reconstruction and modification of spent NCM materials.

EDS, which relies on transmission or scanning transmission electron microscopes, enables the identification of elemental species and quantitative determination of element contents [149,150]. EELS achieves a spatial resolution down to the nanometer or even sub-nanometer scale, making it suitable for fixed-point microanalysis of single particles. Furthermore, this characterization technique enables qualitative and quantitative analysis of Li occupation in NCM lattices and surface Li depletion, while revealing the coordination environments of metal elements and local variations in valence states (Figure 3g) [151,152,153]. TOF-SIMS can simultaneously detect inorganic elements and organic molecules with nanometer-scale depth resolution. Furthermore, it allows quantitative analysis of the gradient of surface lithium loss, the thickness of transition metal dissolution-enriched layers, and the accumulation degree of interfacial organic byproducts in degraded NCM materials (Figure 3h) [154,155]. EXAFS is mainly employed to elucidate local atomic coordination structures, including the coordination number, bond length, and coordination distortion of metal atoms. Meanwhile, it can quantitatively characterize the deterioration of coordination environments at metal sites during lithium deintercalation and cycling processes [156].

#### 3.1.3. Characterization of Three-Dimensional Microstructure and Cross-Section

Three-dimensional microscale damages such as intragranular microcracks, pore evolution, and interfacial voids emerge in NCM during long-term cycling [157,158,159]. Furthermore, such structural defects extend from the particle surface layer to the bulk phase, while 2D planar characterization only acquires information from local cross-sections and fails to fully reconstruct the three-dimensional spatial morphology inside particles. Therefore, ex situ nanoscale characterization techniques focusing on 3D spatial analysis are required. Such techniques encompass Focused Ion Beam (FIB), dual-beam FIB-SEM systems, Nano X-ray computed tomography (nano-XCT), and other related instruments [160,161,162]. They enable a nanoscale 3D characterization system to realize three-dimensional structural visualization and quantitative statistics of single particles and microelectrode regions. Meanwhile, multidimensional information inside particles can be acquired simultaneously, offsetting the limitation of conventional 2D characterization in resolving three-dimensional bulk defects [163]. Most importantly, they act as vital testing approaches to reveal the 3D damage evolution of NCM particles, evaluate the mechanical stability of electrodes, and guide the repair processes of spent ternary cathode materials.

FIB serves as a core tool for controllable cross-section preparation at the nanoscale. This technique artificially exposes flat cross-sections of particles to realize cross-sectional observation of arbitrarily designated microregions [164]. Furthermore, this technique achieves nanometer precision in cross-section fabrication, yet it is categorized as destructive characterization and is commonly utilized as a pretreatment method for TEM and SEM specimens. FIB-SEM enables serial nanoslicing of selected particle regions with simultaneous acquisition of cross-sectional SEM images, allowing quantitative output of 3D structural parameters of materials (Figure 3i) [165,166]. In contrast to conventional FIB, which only provides a single cross-section, FIB-SEM can fully reconstruct the internal structure of individual NCM particles and directly visualize internal structural damage induced by charge–discharge cycling. Nano-XCT enables non-destructive three-dimensional reconstruction via multi-angle projection imaging. It requires no sample destruction and can fully preserve the intrinsic three-dimensional structure of particles [167]. Furthermore, this technique allows direct observation of the internal structure of intact NCM particles and enables large-field three-dimensional imaging of electrodes and composite electrode sheets [168]. Notably, the merits of this characterization technique lie in full non-destructive measurement and a wide field of view, whereas its spatial resolution is inferior to that of FIB-SEM.

In short, the aforementioned ex situ characterization techniques each possess distinctive strengths, and no single characterization method is capable of unraveling the failure modes of NCM materials. Consequently, multi-technique combined characterization strategies are required in practical research to unravel the structural degradation mechanism of NCM at the nanoscale, providing microscopic theoretical guidance for structural regeneration and electrochemical performance improvement of spent ternary cathode materials.

### 3.2. In Situ Dynamic Evolution Characterization Techniques

NCM undergoes continuous crystal evolution behaviors such as reversible layered phase transitions and accumulation of localized lattice strain during Li^+^ intercalation and deintercalation [169,170,171]. Furthermore, the continuously accumulated microscopic lattice distortion during long-term cycling acts as the core inducement for capacity fading and structural pulverization. However, ex situ offline characterization only enables characterization of materials before and after cell disassembly after the completion of charge–discharge cycles [172]. Moreover, such techniques fail to acquire key information on the dynamic crystalline evolution within charge–discharge windows and struggle to clarify the temporal evolution law of crystalline degradation [173]. In contrast, in situ characterization techniques permit real-time tracking of the continuous dynamic evolution of materials during charge–discharge cycling, overcoming the constraint of ex situ characterization that it cannot capture transient structural states [174,175]. Consequently, such techniques serve as core characterization approaches to correlate electrochemical processes with the degradation mechanisms originating from the dynamic evolution of nanomorphology and crystal lattice.

**Figure 4:**
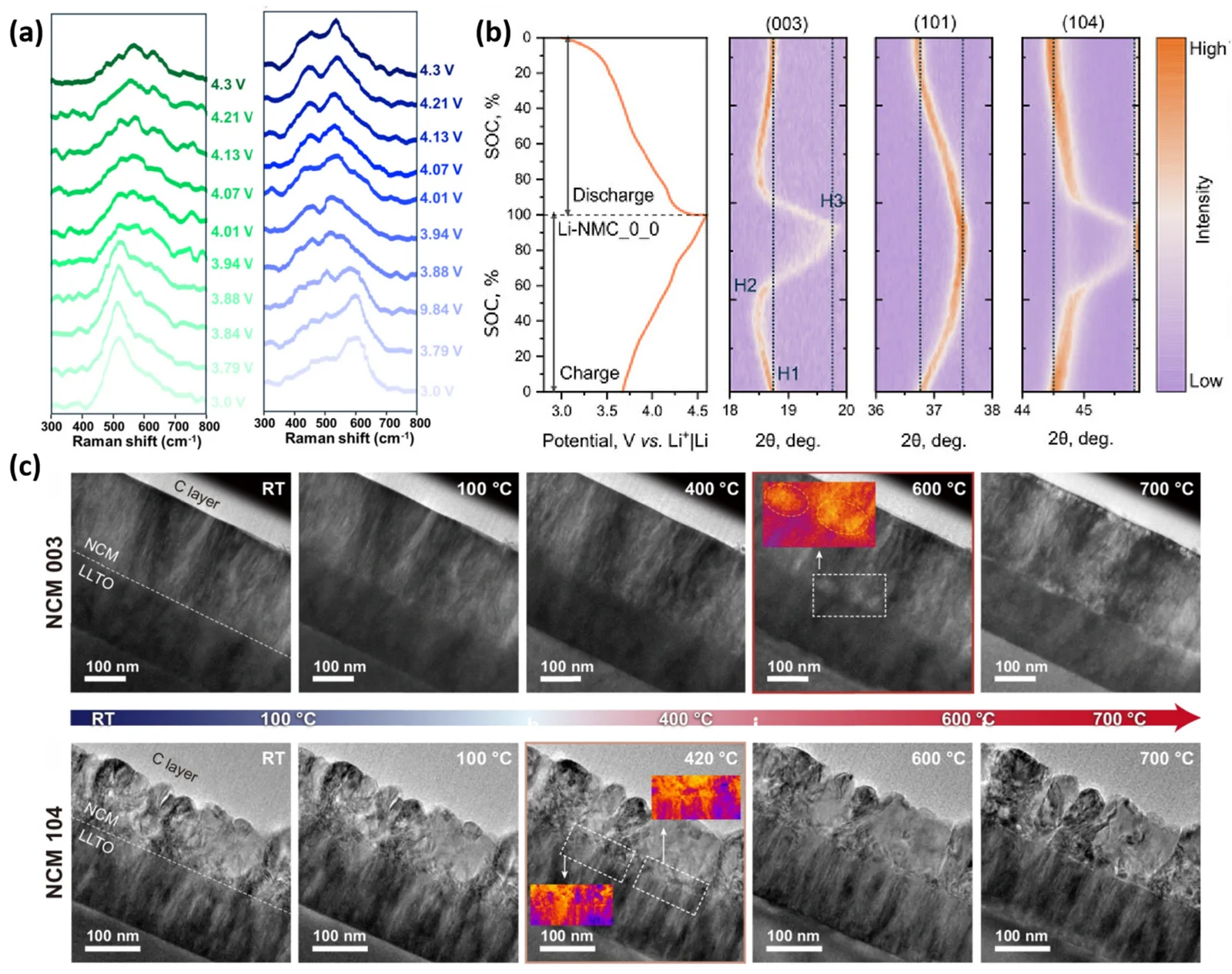
(**a**) In situ Raman spectra during the first charge process [155]. Copyright 2024, John Wiley & Sons, Inc. (**b**) Contour plot of operando XRD data for the Li–NMC_0_0 sample during the first cycle [182]. Copyright 2025, John Wiley & Sons. (**c**) In situ TEM snapshots of NCM003 and NCM104 upon heating, where morphological changes are observed at 600 °C and 420 °C, respectively. The insets are color-coded images within the white dashed boxes, showing the contrast evolution of TEM images during the in situ experiment [153]. Copyright 2024, Springer Nature.

Common in situ characterization techniques include in situ SEM, in situ XRD, in situ TEM, in situ Raman (Figure 4a), and so forth [155,176,177,178]. The resolution of in situ SEM can reach several nanometers, and it allows real-time observation of microstructural evolution [179]. In situ XRD (Figure 4b) enables real-time Rietveld refinement of lattice parameters, unit cell volume, grain size, and microstrain variations. Meanwhile, it can quantitatively track the multiphase phase transitions and the growth trend of diffraction peak intensities of impurity phases during the charge–discharge cycling of NCM [180,181,182]. This technique features compatibility with long-cycle in situ monitoring and a high degree of standardization in data analysis. In situ TEM (Figure 4c) serves as a core in situ characterization technique for dynamically tracking micromorphology at the nanoscale [153,183]. Furthermore, this technique can capture information on transient and intermediate morphological evolution during electrochemical processes. Meanwhile, it simultaneously reveals the dynamic formation mechanisms of NCM particle cracking, surface phase transformation, and the growth of interfacial byproducts [184].

This subsection focuses on typical in situ characterization techniques widely adopted for the failure analysis of NCM cathode materials. Meanwhile, other relevant in situ characterization technologies are also briefly summarized and compiled in Table 1.

In practical research on the failure mechanism of NCM cathodes, ex situ and in situ characterization techniques are commonly combined for joint testing. Furthermore, this integrated strategy enables full-scale tracking of the evolutionary chain ranging from crystal phases to micron-sized particles and atomic-scale interfaces. Meanwhile, it allows a comprehensive elaboration of the multi-stage failure mechanism of NCM materials, thereby furnishing complete and reliable dynamic experimental evidence for the rational design of targeted doping and surface-coating modification strategies.

## 4. Optimization of Structural Stability of NCM Layered Cathodes via Nanoscale Modification Strategies

Multiple failure mechanisms occurring in NCM during cycling constitute the core bottleneck restricting the improvement of energy density for NCM materials [207]. Hence, to effectively address the structural and electrochemical drawbacks of layered NCM cathodes, researchers have carried out investigations focused on nanoscale modification strategies to boost their electrochemical performance. At present, the performance optimization approaches of layered NCM materials primarily fall into two types, namely nanocoating modification [208,209] and elemental doping modification [210,211]. Both strategies work at the nanoscale to precisely improve the structural stability and interfacial properties of the materials. Furthermore, these approaches restrain material degradation at both bulk lattice and surface interface levels to elevate the electrochemical service performance of layered NCM cathodes.

### 4.1. Nanocoating Modification

Nanocoating modification has emerged as one of the prevailing modification strategies to optimize interfacial stability and enhance the electrochemical performance of layered NCM cathodes owing to its good process compatibility and interfacial modulation effect. Nanocoating modification mainly constructs a chemically passivated nanoscale protective layer on the surface of NCM particles to mitigate side reactions of layered NCM cathodes at the solid–liquid interface [212]. Meanwhile, it improves interfacial compatibility and stability, accelerates lithium-ion diffusion kinetics, and prevents surface failure phenomena such as phase transformation and microcrack generation [213,214]. This chapter elaborates on this modification strategy from the perspectives of nanocoating methods and coating species.

#### 4.1.1. Coating Modification Methods

The thickness and uniformity of the coating layer are critical parameters determining the modification effectiveness of coated layered NCM cathodes. Furthermore, coating thickness acts as a pivotal parameter to trade off interfacial protection capacity and charge transport kinetics [215]. Excessively thick microscale and submicroscale coating layers will increase lithium-ion diffusion resistance and electrode interfacial impedance, thereby inducing the reversible specific capacity decay of layered NCM cathodes. Conversely, thin coating layers can suppress interfacial side reactions and accelerate Li^+^ diffusion kinetics, thereby improving the electrochemical performance of layered NCM cathodes [216,217]. In addition, Non-uniform coating layers induce inhomogeneous current distribution and localized stress concentration inside electrodes, thereby accelerating particle degradation and sharp capacity drop. In contrast, uniform coating enables homogeneous dispersion of interfacial stress and consistent Li^+^ transport pathways, simultaneously avoiding the dual drawbacks of exposed defects and redundant coating layers. Therefore, selecting a suitable experimental protocol to fabricate thin and uniform coating layers constitutes one of the optimized strategies for nanocoating modification of layered NCM cathodes.

**Figure 5:**
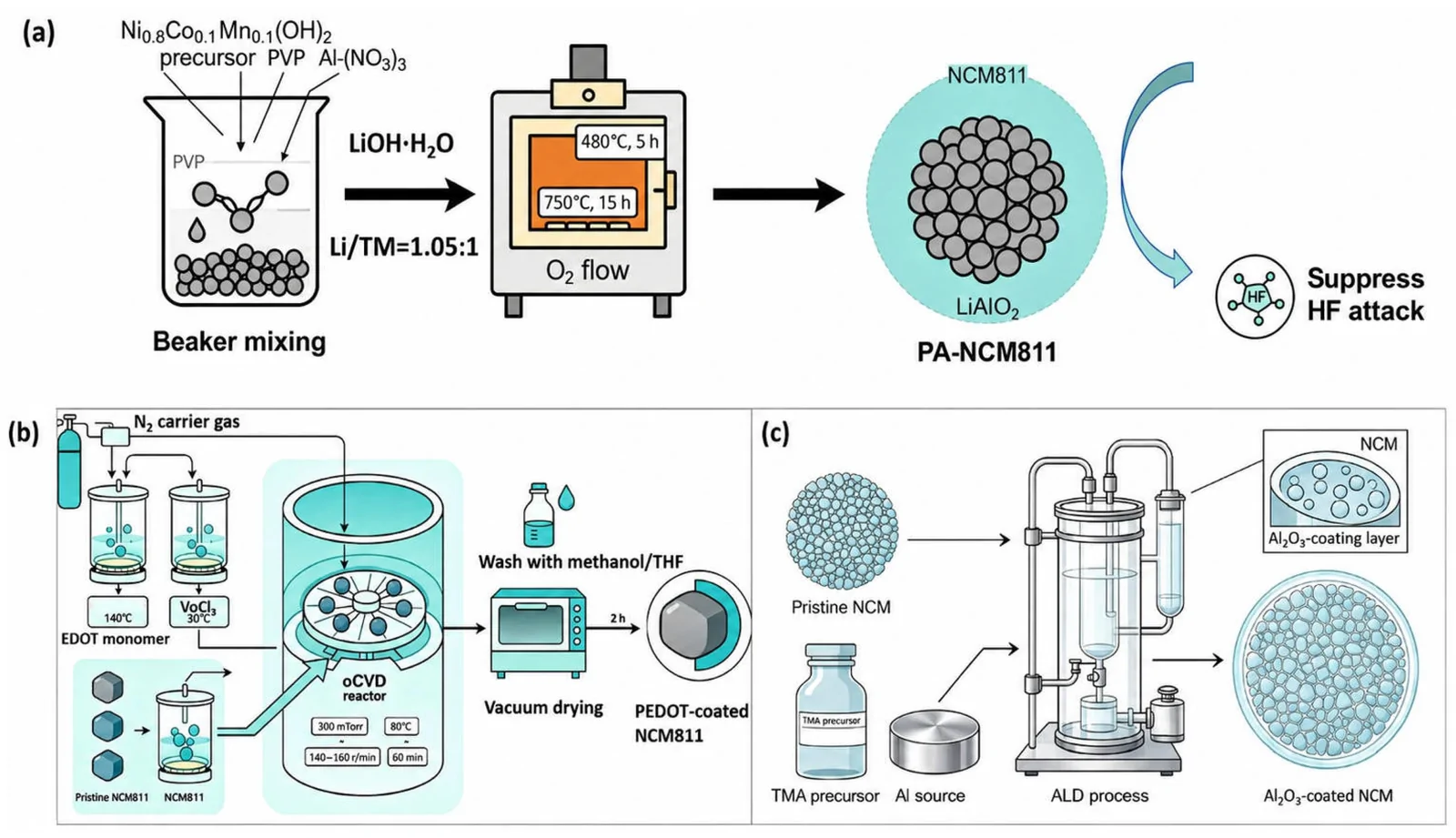
(**a**) Schematic flow chart of modification by acid etching method; (**b**) schematic illustration for the preparation of PEDOT-coated NCM811 via oCVD method; (**c**) schematic of the fabrication of NCM@xAl by ALD.

Liquid-phase coating modification represents a mainstream wet-chemical surface modification strategy. Such approaches generally operate under mild reaction conditions, enabling the fabrication of uniform coatings within low-temperature liquid environments. Furthermore, precursors and modified ions can be homogeneously dispersed in liquid-phase systems, which allows precise regulation of coating components and compatibility with diverse coating material systems. Li et al. [218] employed the sol–gel method to construct an amorphous fast ionic conductor Li_1.5_La_1.5_TeO_6_ (LLTeO) coating with a thickness of 5 nm on the surface of LiNi_0.83_Co_0.12_Mn_0.05_O_2_ (NCM83). This coating can reduce residual lithium species on the NCM surface, thereby effectively suppressing interfacial side reactions and accelerating Li^+^ diffusion kinetics. Although thin nanocoatings fabricated via this strategy exhibit favorable adhesion, the viscosity of the sol is difficult to regulate; the calcination process tends to damage the crystal lattice of particles, and uneven coating distribution is prone to occur. To avoid damage to the crystal structure of NCM materials during calcination, Zhang et al. [219] fabricated a CeO_2_ coating with a thickness of approximately 20 nm on the surface of LiNi_0.9_Co_0.05_Mn_0.05_O_2_ (NCM9005) via liquid-phase deposition. Meanwhile, Ce ions are also doped into the interior of crystal grains. In addition, the CeO_2_ coating can effectively suppress interfacial side reactions on the cathode side. Meanwhile, the high dissociation energy of Ce–O bonds generated by Ce doping enhances the structural stability of the bulk lattice, alleviates Li/Ni cation mixing, and broadens the transport channels for lithium ions. This strategy features a straightforward procedure and enables the preparation of uniform coatings with tunable thickness. However, this strategy suffers from a low deposition rate and is incapable of realizing multi-element co-doping. In addition, Wang et al. [220] developed a strategy combining acid etching and polymer crosslinking to construct protective coatings (Figure 5a). This method can form a uniform LiAlO_2_ coating with a thickness of approximately 10 nm on the surface of LiNi_0.8_Co_0.1_Mn_0.1_O_2_ (NCM811). Meanwhile, the as-formed coating efficiently restrains lattice oxygen release and shields NCM811 from HF corrosion. Nevertheless, this route involves complicated procedures and is limited in industrial-scale application.

Although liquid-phase coating modification is capable of producing uniform nanoscale thin coatings, such approaches suffer from high manufacturing costs, heavy wastewater treatment burdens, and poor scalability for mass production. In contrast, dry-state coating modification is a surface modification technique that introduces no solvents and achieves surface functionalization merely through mechanical interaction between solid powders. Such strategies feature concise procedures, eco-friendliness, low energy consumption, high mass-production efficiency, as well as low modification cost for industrial application. Among them, the high-speed dry mixing coating technique is a widely adopted dry modification method for industrial-scale production. Xiang et al. [221] adopted this technique to uniformly coat Ketjen Black on the surface of NCM811 with a thickness of approximately 6 nm. Furthermore, the coating can accelerate electron transport and form three-dimensional electron-migration pathways when combined with carbon nanotubes. Nevertheless, the coating obtained by this method exhibits unsatisfactory uniformity and tends to agglomerate and peel off easily. To fabricate thin and uniform coating layers, Liu et al. [222] innovated an oxidative chemical vapor deposition (oCVD) system. Meanwhile, an ultra-thin PEDOT coating with a thickness of approximately 10 nm was deposited on the surface of NCM811 particles via this technique (Figure 5b). Furthermore, the coating can effectively avoid undesired phase transitions during high-temperature aging, as well as suppress the propagation of grain-boundary cracks and lattice oxygen release. However, this approach incurs high operating costs and is only applicable to fundamental laboratory research. In addition, Atomic Layer Deposition (ALD) allows precise control over coating thickness and morphology, and enables uniform deposition of atomic-scale thin layers on substrates with complex surfaces [223]. Zhao et al. [224] utilized this technique to fabricate a dense, uniform Al_2_O_3_ coating on the surface of LiNi_0.5_Co_0.2_Mn_0.3_O_2_ (NCM523) (Figure 5c). Studies reveal that the Al_2_O_3_ coating inhibits TM leaching from NCM and thus prevents the occurrence of the TM shuttle effect. Furthermore, the coating preserves the surface integrity and structural stability of NCM, endowing the material with superior cycling durability and rate capability. This method enables the formation of fully covered nanoscale coatings, yet it suffers from high costs in mass production.

Although the above strategies can form coating layers and improve the electrochemical performance of NCM, each suffers from certain drawbacks. Therefore, selecting an appropriate experimental scheme according to the research objectives exerts a remarkable influence on strengthening the modification effect and improving experimental efficiency.

#### 4.1.2. Elements for Coating Modification

In terms of this coating strategy, apart from diverse synthetic routes, the selected coating elements also show obvious discrepancies. Nanocoatings derived from different elements exhibit distinct performances in isolating NCM particles from electrolyte contact, suppressing interfacial side reactions, and improving the electrochemical properties of batteries.

Benefiting from outstanding structural stability, metal oxide coating layers can effectively regulate the internal crystal structure of materials and suppress Li/Ni cation mixing, grain boundary migration, and the diffusion of TM ions [225]. Meanwhile, it stabilizes the framework structure of the NMC matrix, thereby mitigating electrode structural degradation fundamentally and remarkably improving the cycling stability and electrochemical reversibility of the material [226]. Recent studies reveal that coatings composed of high-valence elements (e.g., Ta^5+^, Sb^5+^, Nb^5+^, and W^6+^) can effectively tailor the microstructure of concentration-gradient (CG) NMC materials [227,228]. Savina et al. [229] introduced Ta_2_O_5_ into CG-NMC9 (LiNi_0.9_Mn_0.067_Co_0.033_O_2_ featuring concentration gradients). Further investigations reveal that a tantalum-rich surface layer with a thickness of around 5 nm can be formed on the material. Meanwhile, the concentration-gradient structure and elongated primary particles of NMC9 are well preserved. Furthermore, the coating can inhibit Li/Ni cation disorder and TM ion diffusion. Nevertheless, single metal oxide coatings possess low lithium-ion conductivity, so auxiliary optimization by combining other materials or structures is required. As a new family of porous materials, metal–organic frameworks (MOFs) are assembled using metal nodes and organic linkers as structural building units. Numerous studies have confirmed that their distinctive pore structure can positively improve the performance of coating modification [230,231]. To address this issue, Li et al. [232] proposed a zirconia coating derived from zirconium-based metal–organic frameworks. This strategy constructs a uniform amorphous ZrO_2_ coating with a thickness of approximately 3 nm on the surface of LiNi_0.6_Co_0.2_Mn_0.2_O_2_ (NCM622). Furthermore, the coating can not only effectively suppress harmful interfacial side reactions and stabilize the interfacial structure, but also mitigate lattice oxygen release and accelerate the transport kinetics of interfacial Li^+^.

**Figure 6:**
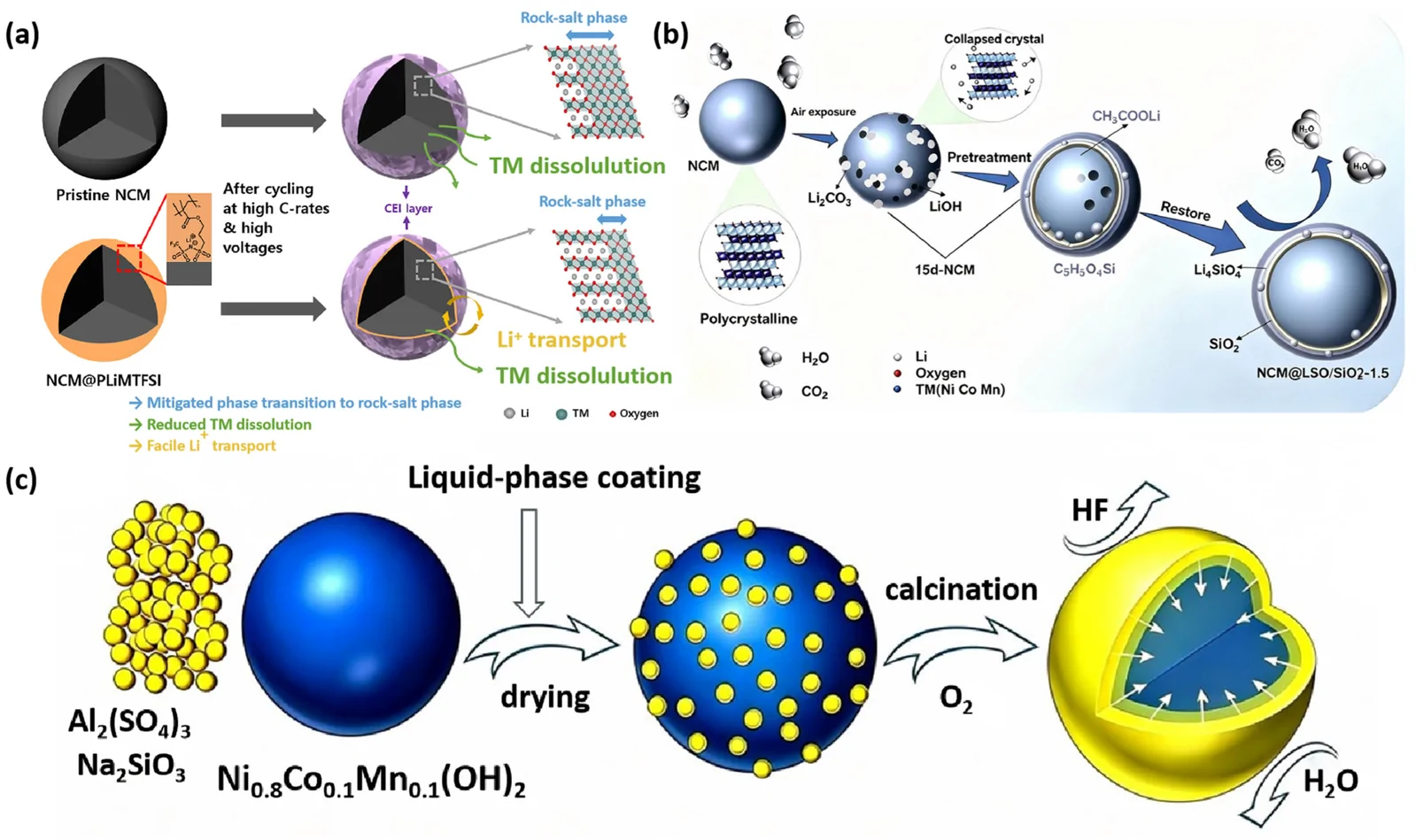
Schematic diagrams of different modification mechanisms and preparation processes. (**a**) The modification mechanism of PLiMTFSI coating for NCM secondary particles [236]. Copyright 2022, John Wiley & Sons. (**b**) Structural deterioration issues and corresponding modification repair strategies of NCM electrodes. (**c**) Synthetic route for NCM811 modified by Al_2_(SO_4_)_3_ and Na_2_SiO_3_.

Inorganic–organic polymer coatings can construct flexible and highly compatible interfacial layers [233]. Furthermore, such coating layers can optimize the interfacial structure of cathodes, effectively buffer volume variation during electrode charging and discharging, and restrain the initiation and propagation of microcracks in particles [234,235]. Kim et al. [236] found that a new-type LiMTFSI-based polymer coating enhances the electrochemical performance of NCM811 (Figure 6a). Meanwhile, the uniform coating, possessing a thickness of nearly 20 nm, can suppress the transformation toward the Rock-Salt phase on the material surface, alleviate the dissolution of transition metals, and accelerate interfacial Li^+^ conduction. However, organic coating layers suffer from insufficient electrochemical and mechanical stability. To fabricate a coating layer with high mechanical strength, low interfacial impedance, uniform morphology, and strong interfacial adhesion, Liu et al. [237] incorporated inorganic reinforcing agents into a polymer matrix to form an organic–inorganic hybrid polymeric network. The research team introduced titanium oxo clusters (TOCs) to crosslink polyurethane, generating an ~8 nm-thick PHM-T coating on the surface of NCM811. This coating layer acts as an artificial CEI, which effectively mitigates parasitic side reactions of electrolytes and suppresses the generation of microcracks within particles, thereby remarkably enhancing the structural stability and rate capability of NCM cathodes.

Coatings composed of lithium salts and phosphates improve the ionic conductivity of NCM and prevent electrolyte corrosion. Meanwhile, such coatings can in situ consume residual lithium impurities on material surfaces, suppress TM migration and interfacial side reactions, and simultaneously achieve high ionic conductivity and structural protection [238,239]. He et al. [240] adopted Li_4_SiO_4_/SiO_2_ as structural regulators to fabricate a nanocoating on the surface of NCM particles (Figure 6b), which separates NCM materials from the electrolyte and thus effectively suppresses electrolyte corrosion. In addition, Gupta et al. [241] deposited a uniform Li_3_PO_4_ coating with a thickness of 13.6 nm on NCM811 surfaces. This coating not only possesses Li^+^ conductivity but also maintains the structural integrity of NCM, suppresses interfacial reactions, and improves the cycling performance of lithium-ion batteries. Shadab [242] et al. discovered that LiH_2_PO_4_ enables bifunctional modification. It can not only directly remove residual lithium on NCM surfaces but also convert these residues into a ~4 nm-thick Li_3_PO_4_ coating in situ. Such coating markedly suppresses nickel migration and alleviates electrolyte-triggered side reactions, therefore improving the structural stability of layered cathodes.

In addition to modifying NCM via single-component coatings, multi-functional composite interphases co-constructed by two or more materials can consume residual lithium on NCM particle surfaces and resist corrosion induced by HF. These composite layers effectively improve the thermal stability and electrochemical performance of batteries, adapt to harsh operating conditions such as high temperature and high voltage, and significantly expand the application scope of cathode materials [243,244]. To alleviate structural degradation of NCM cathodes under high operating voltage and elevated temperature conditions, Xiong et al. [245] co-utilized LiAlO_2_, LiBO_2_, Al_2_O_3_, and B_2_O_3_ to construct an 18 nm-thick multifunctional composite interphase on single-crystal NCM811 surfaces. This composite layer consists of fast ionic conductor phases of α-LiAlO_2_ and LiBO_2_, while the byproducts γ-Al_2_O_3_ and B_2_O_3_ act as composite protective layers. Rapid lithium-ion transport channels exist within the ionic conductor layer, which can accelerate the diffusion rate of Li^+^. The composite protective layer can effectively suppress irreversible phase transitions and oxygen evolution reactions. This multifunctional composite interphase delivers protective effects under high-temperature and high-voltage conditions and facilitates the formation of uniform thin films. In addition, He et al. [246] employed LiAlO_2_ and Li_2_SiO_3_ to form an uneven dual coating with thickness ranging from 50 to 250 nm on NCM811 surfaces (Figure 6c). By constructing three-dimensional lithium-ion diffusion pathways and a surface protective film, this coating improves the lithium-ion diffusion coefficient and electronic conductivity of AS-NCM811 (LiAlO_2_ and Li_2_SiO_3_ serve as dual-coating agents for NCM811). Furthermore, AS-NCM811 exhibits outstanding cycling performance over the temperature range from room temperature to 55 °C.

Four categories of coating systems, namely metal oxides, nonmetallic and organic–inorganic hybrids, lithium salts/phosphates, and multi-material composites, can effectively restrain Li/Ni cation mixing, transition metal ion diffusion, and grain boundary migration, as well as the propagation of intra-particle microcracks, relying on the structural stability of coating layers, thereby remarkably boosting the cycling stability of cathode materials.

### 4.2. Doping Modification at Nanoscale

Nanoscale doping modification represents another mainstream strategy to enhance the structural stability and optimize the electrochemical performance of NCM cathodes. Nanocoating modification primarily relies on coating layers to isolate interfacial reactions between NCM and electrolyte, thereby enhancing the electrochemical performance and structural stability of NCM cathodes. In contrast, nanoscale doping introduces foreign elements into the interior of grains to reconstruct, optimize, and stabilize the crystal framework of NCM materials starting from the lattice level. Furthermore, this strategy strengthens the stability of the layered structure, suppresses lattice oxygen loss, and mitigates lattice distortion, microcrack generation, and Rock-Salt phase formation [247,248,249]. Doping modification can be classified into anion doping, cation doping, and multi-ion co-doping according to the types of doped ions.

#### 4.2.1. Anion Doping

Anion doping employs nonmetallic anions as dopants to substitute the intrinsic oxygen anions in the host lattice, which serves as a regulation strategy for the anion sublattice. Further, its core functions include tuning the band structure, creating anion vacancies, and optimizing charge-transfer efficiency as well as surface reaction activity. Ahn et al. [250] verified that moderate F^−^ doping can remarkably enhance the electrochemical performance of NMC9055. Related studies reveal that F substitution alleviates cation mixing, hinders oxygen sliding along the a–b plane, and suppresses the H2–H3 phase transition of NMC under high voltage, thereby stabilizing the crystal structure and improving Li^+^ migration kinetics. However, anion doping tends to generate excessive anion vacancies, which trigger slight collapse of the lattice framework and degrade the cycling durability of cathode materials. To further boost the modification efficacy, researchers have discovered that constructing an elemental concentration gradient inside cathode particles can drastically reduce internal phase inhomogeneity and improve specific capacity [251]. To improve the electrochemical performance of NCM cathodes, Li et al. [252] introduced boron-based polyanions via gradient engineering and surface lattice regulation to fabricate a gradient doping structure with boron-based polyanions. In addition, this treatment triggers surface lattice reconstruction and generates a stable Rock-Salt-free protective layer. With this doping structure, stress accumulation originating from phase transitions can be effectively alleviated, and the structural stability of the material is enhanced. Simultaneously, the protective layer efficiently blocks side reactions between the cathode surface and electrolyte.

#### 4.2.2. Cation Doping

**Figure 7:**
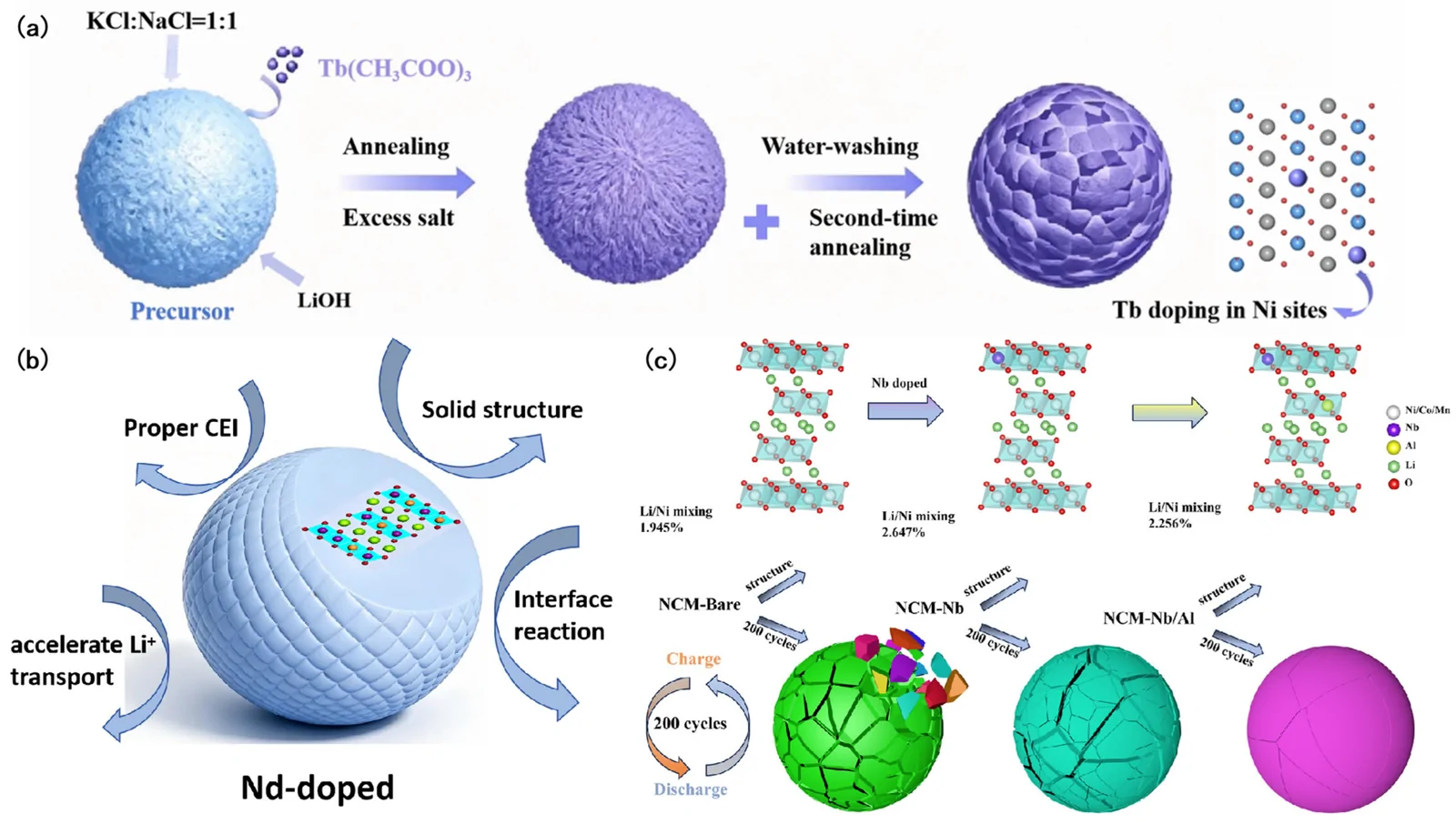
(**a**) Schematic of the synthesis procedure for Mo-doped NCM materials. (**b**) Schematic illustration of performance evolution mechanisms for Nd-modified NCM9055. (**c**) Schematic of doping sites and failure mechanism of Nb/Al co-doping [259]. Copyright 2025, John Wiley & Sons.

Cation doping realizes modification by introducing aliovalent or isovalent metal cations into the host lattice to substitute original cation sites of the matrix. This strategy can tune lattice parameters and local electronic structures, effectively enhance electronic conductivity and structural stability of materials, and restrain lattice phase transitions as well as cation mixing defects. However, its modification dimension is limited, and it can only optimize the performance of the cation sublattice and hardly simultaneously ameliorate defects related to anion coordination and interfacial properties. Jian et al. [253] demonstrated that Tb^4+^ doping can effectively boost the rate capability and structural stability of SNCM (Figure 7a). Relevant studies reveal that Tb^4+^ uniformly substitutes Ni sites, accomplishes charge compensation through the reduction in Ni^3+^ to Ni^2+^, reduces the valence state of Ni, and induces the generation of Li vacancies. As a result, the Li^+^ transport kinetics within layered oxides are greatly enhanced, and the H1–H2 phase separation is eliminated. However, when addressing the damage of layered structures induced by accumulated lattice strain, existing modification strategies generally overlook the release of lattice oxygen. To address this issue, Jiang et al. [254] confirmed that Nd^3+^ doping can effectively suppress unit cell volume variation and hinder the formation of oxygen vacancies (Figure 7b). Experimental results reveal that the (003) interplanar spacing of doped NCM increases by 0.006 nm, which facilitates accelerated Li^+^ transport. Meanwhile, a uniform CEI film with a thickness of 2 nm forms on the grain surface, efficiently restraining lattice oxygen loss. In addition, Luo et al. [255] proposed a two-step Nb modification strategy to achieve gradient Nb doping with a dense outer region and sparse inner distribution in NCM622. In particular, low-concentration Nb doping in the interior can enlarge interplanar spacing, accelerate lithium-ion diffusion, and strengthen plane stability, thus effectively alleviating the performance degradation of the material. Then, the dense distribution of Nb on the particle surface can not only suppress interfacial side reactions but also improve the electronic conductivity of the material. Furthermore, Nb doping induces radial elongation of grains, which relieves internal stress during phase transformation and shortens the diffusion path of Li^+^. However, doping with a single type of cation tends to induce concentrated lattice stress and uneven local lattice distortion, making it difficult to balance the overall comprehensive performance of the material.

#### 4.2.3. Co-Doping

Co-doping specifically refers to multi-ion co-doping, a modification strategy that simultaneously introduces two or more ions with different radii and valence states to jointly substitute lattice sites of the host material. In recent years, dual-ion and even multi-ion doping modification technologies have attracted extensive attention from researchers [256,257]. This approach can precisely regulate lattice distortion and optimize the local electronic structure through the synergistic effect of dual ions or multi-ions, compensating for the limited modification effect of single-ion doping. Meanwhile, it inhibits the imbalance of lattice stress and achieves the synergistic improvement of electrical conductivity and structural stability of the material. Shen et al. [258] adopted an Al^3+^ and Zr^4+^ dual-cation doping strategy and found that Al–Zr co-doping alleviates Li^+^/Ni^2+^ mixing. Consequently, Li^+^ diffusion kinetics are improved, and the coexistence of adverse phase transformations is restrained. In addition, Al–O and Zr–O bonds can strengthen the stability of the lattice oxygen framework and restrain detrimental phase transitions as well as lattice oxygen loss, thus improving the structural stability of NCM. In addition, Ma et al. [259] found that there exists a mutually complementary effect between Nb^5+^ and Al^3+^ dopants (Figure 7c). Studies indicate that Nb^5+^ doping effectively restrains the expansion of primary particles, which accelerates Li^+^ diffusion and increases the density of the crystal structure, yet it aggravates Li^+^/Ni^2+^ cation mixing. However, the introduction of Al^3+^ effectively alleviates the adverse effects induced by Nb^5+^ doping and reduces electrode polarization during long-term cycling. The synergistic effect of Nb^5+^/Al^3+^ co-doping remarkably improves the layered structure integrity and electrochemical performance of high-nickel NCM cathodes.

## 5. Conclusions and Outlook

This review summarizes the nanoscale structural failure mechanisms of NCM cathodes and highlights advanced characterization techniques and corresponding optimization strategies for failure mitigation. The nanoscale failure mechanisms of NCM cathodes include lattice distortion and layered-structure instability at the lattice level, nanocrack generation at the particle level, as well as nanomaterial migration and degradation induced by side reactions at the interface level. Furthermore, special emphasis is placed on analyzing nanoscopic microfailure mechanisms such as interlayer sliding and slide evolution, lattice rotation, and irreversible phase transitions. Subsequently, advanced characterization techniques applicable to analyzing the aforementioned failure mechanisms are further summarized. Special emphasis is placed on the characterization methods, including HRTEM, TOF-SIMS, and XCT, as well as dynamic characterization techniques such as in situ XRD and in situ TEM. Finally, two modification strategies, namely coating and doping, are discussed in detail. The coating modification strategy is discussed from two perspectives: coating methods and coating species. Additionally, doping modification strategies are summarized from three routes: cation doping, anion doping, and co-doping, with special focus on the influences of various dopant ions on NCM cathodes.

**Figure 8:**
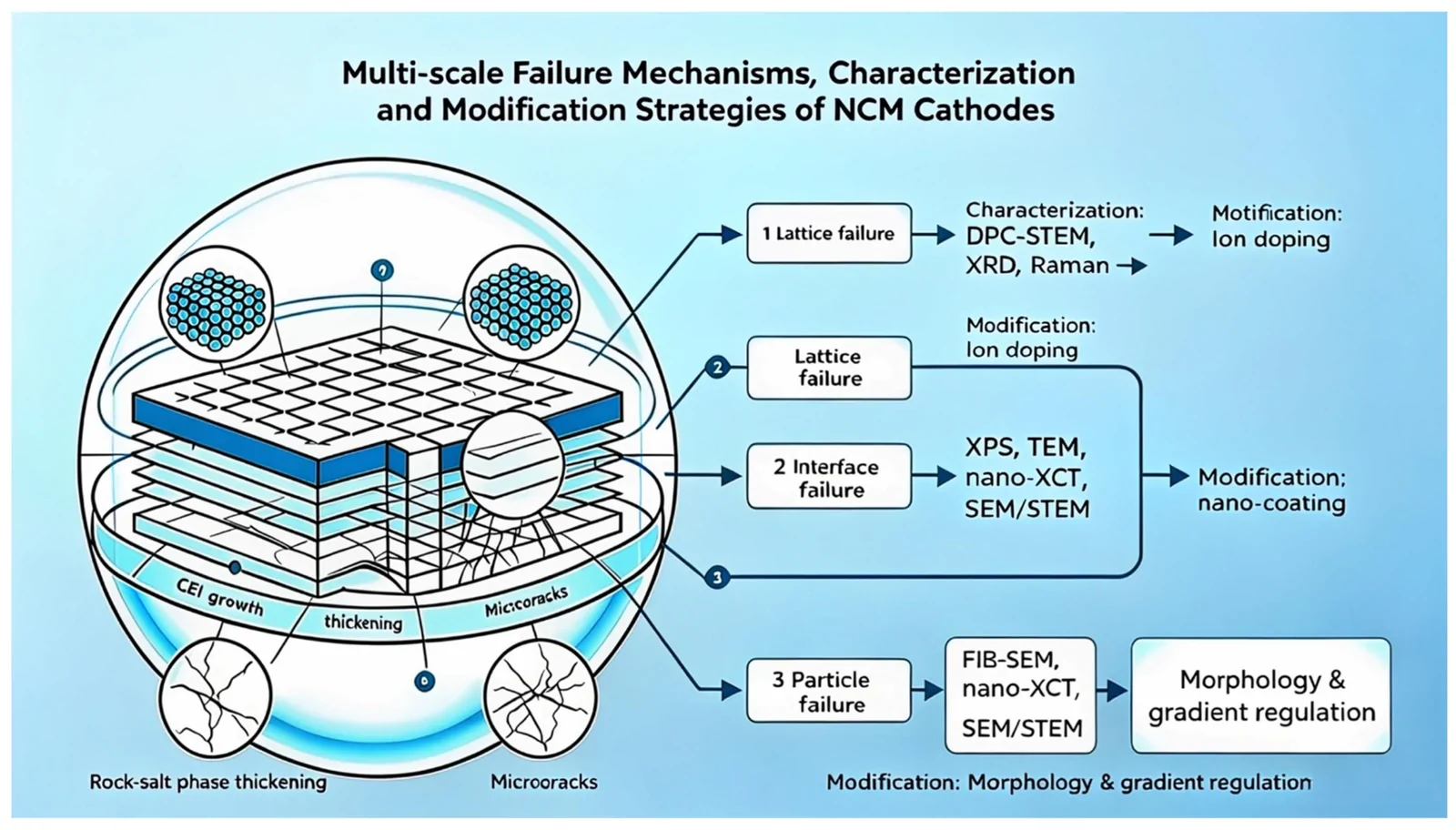
Schematic illustration of multi-scale failure mechanisms occurring in layered NCM cathode particles, together with corresponding characterization techniques and targeted modification strategies.

The aforementioned research framework of analyzing NCM degradation, designing modification strategies, and conducting supporting precise characterizations has evolved into the mainstream research direction in the field of ternary cathode materials (Figure 8). However, it should be pointed out that there remain urgent challenges to be addressed in this field.

(1) Scale-up modification toward industrialization. Modification and future upcycling processes are of great significance for the industrial preparation of NCM materials. However, this technology still faces numerous substantial challenges at the present stage. Most existing studies are restricted to lab-scale preparation with a batch output of only tens of grams. Meanwhile, multiple factors remain bottlenecks for the large-scale upcycling of NCM materials. On one hand, several feasible laboratory-scale upcycling protocols are difficult to implement in industrial production lines. On the other hand, integrated continuous mass production processes have not yet been fully developed, resulting in low efficiency for batch fabrication.
(2) Coupled research system of in situ multi-field nanocharacterization and molecular dynamics simulation. Current laboratory investigations into the mechanisms of NCM materials mostly rely on ex situ characterizations, which makes it difficult to clarify the underlying kinetic laws. Subsequent research shall deeply integrate in situ multi-field nanocharacterization and molecular dynamics simulation to realize bidirectional coupling. On the one hand, dynamic evolution data can be acquired in real time to supply actual working-condition boundary conditions and experimental calibration references for simulation models. On the other hand, molecular dynamics simulations can compensate for the limitations of characterizing ultramicroscopic transient reactions, quantitatively revealing the intrinsic correlation between microstructural defects and electrochemical performance.
(3) Coupling system of coating-doping knowledge graph and machine learning. Existing laboratory research still suffers from blindness in the design of modification strategies, which leads to low experimental efficiency. In the future, precise nano-modification systems can be developed based on the evolution laws of atomic-scale defects, accompanied by the construction of knowledge graphs and the introduction of machine learning algorithms. This strategy can effectively eliminate blind trial and error during the selection of modification elements, rapidly screen optimal modification routes, and thus improve experimental efficiency.

## Figures and Tables

**Table 1:** Summary of functions of in situ characterization techniques on NCM cathodes.

In situ Characterization Techniques	Fields of Characterization on NCM Cathodes	Ref.
In situ TEM	Irreversible phase transition; generation of microcracks; growth of CEI film; interlayer sliding and gliding	[185,186]
In situ SEM	Morphology and volume evolution of NCM particles; crack propagation; growth of cathode–electrolyte interphase (CEI) film	[179,187]
In situ XRD	Variation in lattice parameters of NCM; irreversible phase transition; degree of cation mixing	[188,189]
In situ FT-IR	Evolution of chemical compositions of cathode–electrolyte interphase (CEI) film; lattice oxygen release; interfacial side reactions	[190,191]
In situ EIS	Impedance evolution during charge–discharge processes; diffusion kinetics of lithium ions; characterization of CEI film growth	[192,193,194]
In situ Raman	Dynamic lattice evolution of NCM; irreversible phase transition; dynamic evolution of cation mixing	[195,196,197]
In situ XANES	Valence state evolution of TM elements; dissolution of TM species and their interfacial deposition behavior	[198,199]
In situ TXM	Local microscopic phenomena at electrode interfaces	[200,201,202]
In situ CT	Initiation and three-dimensional propagation of cracks; structural evolution of three-dimensional pores	[203,204]
In situ NMR	Quantify the irreversible lithium loss; reflect lattice distortion	[205,206]

## Data Availability

No new data were created or analyzed in this study. Data sharing is not applicable.

## Abbreviations

NCM	LiNi_x_Co_y_Mn_z_O_2_
Li^+^	Lithium ions
TM	Transition metal
MCRC	Multicrystal Rocking Curve
DCR	Defect chain reactions
SRL	Surface reconstruction layer
CEI	Cathode–electrolyte interphase
2D	Two-dimensional
3D	Three-dimensional
TEM	Transmission electron microscopy
HRTEM	High-resolution transmission electron microscopy
FFT	Fast Fourier transform
EDS	Energy-Dispersive X-ray Spectroscopy
EELS	Electron Energy-Loss Spectroscopy
TOF-SIMS	Time-of-Flight Secondary Ion Mass Spectrometry
EXAFS	Extended X-ray Absorption Fine Structure
FIB	Focused Ion Beam
Nano-XCT	X-ray nano-computed tomography
RT	Room temperature
LLTeO	Li_1.5_La_1.5_TeO_6_
oCVD	Oxidative chemical vapor deposition
CG	Concentration-gradient
MOFs	Metal–organic frameworks
TOCs	Titanium oxo clusters
SC-NCM88	Single-crystal LiNi_0.88_Co_0.09_Mn_0.03_O_2_
ADF-STEM	Annular Dark-Field Scanning Transmission Electron Microscopy
HAADF-STEM	High-Angle Annular Dark-Field Scanning Transmission Electron Microscopy
DPC-STEM	Differential Phase-Contrast Scanning Transmission Electron Microscopy
NCM83	LiNi_0.83_Co_0.12_Mn_0.05_O_2_
NCM9005	LiNi_0.9_Co_0.05_Mn_0.05_O_2_
NCM811	LiNi_0.8_Co_0.1_Mn_0.1_O_2_
ALD	Atomic Layer Deposition
NCM523	LiNi_0.5_Co_0.2_Mn_0.3_O_2_
CG-NMC9	LiNi_0.9_Mn_0.067_Co_0.033_O_2_ featuring concentration gradients
NCM622	LiNi_0.6_Co_0.2_Mn_0.2_O_2_
AS-NCM811	LiAlO_2_ and Li_2_SiO_3_ serve as dual-coating agents for NCM811
